# Femtosecond Laser Nano/Micro Textured Ti6Al4V Surfaces—Effect on Wetting and MG-63 Cell Adhesion

**DOI:** 10.3390/ma12132210

**Published:** 2019-07-09

**Authors:** Georg Schnell, Susanne Staehlke, Ulrike Duenow, J. Barbara Nebe, Hermann Seitz

**Affiliations:** 1 Microfluidics, Faculty of Mechanical Engineering and Marine Technology, University of Rostock, Justus-von-Liebig Weg 6, 18059 Rostock, Germany; 2 Deptartment of Cell Biology, University Medical Center Rostock, Schillingallee 69, 18057 Rostock, Germany; 3 Deptartment Life, Light & Matter, University of Rostock, Albert-Einstein-Str. 25, 18059 Rostock, Germany

**Keywords:** Ti6Al4V, femtosecond laser, microstructure, osteoblasts, morphology, actin cytoskeleton

## Abstract

Nano- and microstructured titanium surfaces have recently attracted attention in the field of regenerative medicine because of the influence which surface characteristics such as roughness and wettability can have on cellular processes. This study focuses on the correlation of surface properties (wettability and nano/micro texture) of laser-structured Ti6Al4V samples with pronounced cell adhesion. Samples were structured with multiple laser parameters in order to create a range of surface properties. Surface characterization was performed by contact angle measurements 1 and 7 days after laser processing. The arithmetic mean roughness of the material surface in an area (Sa) was determined by means of confocal laser scanning microscopy (CLSM). Immediately after wettability tests of the laser-structured surfaces, in vitro experiments with human MG-63 osteoblasts were carried out. For this purpose, the cell morphology and actin cytoskeleton organization were analyzed using CLSM and scanning electron microscopy. On rough microstructures with deep cavities, the cell growth and spreading were inhibited. An improved cellular adhesion and growth on nanostructured and sinusoidal microstructured surfaces could be demonstrated, regardless of hydrophilicity of the surfaces.

## 1. Introduction

The modification and optimization of the surface properties of implants with a focus on cell adhesion was investigated by many research groups [1,2,3,4]. Titanium and titanium alloys are widely used as biomaterial for implants due to their high biocompatibility [5,6]. Specifically, Ti6Al4V material is used for bone implants [7,8], as hip and knee joints, dental and jaw implants, and generally in reconstructive surgery. Surface modification strategies are used to induce bone healing process and osseointegration capability [9]. 

The laser structuring of Ti6Al4V samples is of particular interest for a multitude of biomedical applications [10]. Due to the high reproducibility, flexibility, and capability of generating a wide range of surface structures, femtosecond laser irradiation is a highly attractive manufacturing method to vary the wetting properties [11,12] or cell behavior patterns [13,14]. Therefore, the structuring of separate areas of the implant surface can be used to cause desired and particular cell behavior [15,16]. It can also provide an improved bone-implant interface anchorage [17] or the reduction of bacterial adhesion and biofilm formation [18,19].

It is known that surface characteristics such as roughness and wettability influence cellular processes like spreading, proliferation, differentiation, and intracellular signaling via Ca^2+^ ions [2,3,15]. Often a hydrophilic wetting state of surfaces is associated with an increase of bone formation in the peri-implant region in comparison to hydrophobic surfaces [4]. However, especially on laser structured metallic surfaces, a strong change of the wetting state over time is observed [11,20] and the correlation between hydrophilicity and pronounced cell adhesion is put into question [21]. In summary, the effect of a changed wetting state on cell behavior is unclear and the significance of the correlation between hydrophilicity and pronounced cell adhesion should therefore be analyzed. The aim of this study is to investigate the adhesion, morphology, and growth of human osteoblasts on femtosecond laser produced nano- and microstructured surfaces dependent on the wetting state over time.

## 2. Materials and Methods

### 2.1. Materials and Sample Pretreatment

The experimental investigations were carried out with Ti6Al4V samples (1 × 1 cm). Water-jet cut plates were purchased from S + D Spezialstahl Handelsgesellschaft mbH (Stelle, Germany). The specification complies with the requirements of AMS4911 [22] and WL 3.7164 Part 1 [23]. For a consistent and fine roughness of the surface, the plates were polished with silicon carbide abrasive sandpaper from P320 (t1 = 4 min), P600 (t2 = 4 min) to P1200 (t3 = 8 min) grain size and certain periods and under ultrapure water (Reference). The abrasive treatment was followed by a cleaning in an ultrasonic bath Sonorex Super RK 100/K (Bandelin electronic GmbH&Co.KG, Berlin, Germany) with ultrapure water for 10 min. Drying was performed with dust free cloth and compressed air.

### 2.2. Micromachining

For laser structuring of Ti6Al4V, the femtosecond fiber laser of the type UFFL_60_200_1030_350_SHG of the manufacturer Active Fiber Systems GmbH (Jena, Germany) was used. It is a fiber laser with an amorphous glass with an Yb-doped core. The wavelength used was 1030 nm with linear polarized light and the pulse duration was 300 fs. For deflecting the laser beam, a scanner system of the type intelliSCANse (Scanlab GmbH, Puchheim, Germany) was used. The beam was focused by an F-theta lens with a focal length of 163 mm resulting in a beam spot diameter of about 36 microns. The laser system enables a repetition rate from 50.3 kHz up to 18.6 MHz up to an average power of 60 W. The laser is integrated in the micromachining device Microgantry GU4 (Kugler, Salem, Germany). To create widely different femtosecond laser-induced periodic structures (FLIPPS1 and FLIPPS2) and microstructures (Micro1 and Micro2) with various roughness, the laser parameters were varied in pulse energy, line overlap and procedure of laser passage (line and grid), as shown in Table 1. The pulse overlap was kept constant at 50% and number of overscans was 50. For each structure, two specimens were manufactured. Samples without micromachining were used as references (plane Ti6Al4V specimen).

### 2.3. Surface Characterization

The average area surface roughness (Sa) and the elevation profile (depth and width of the resulting pillars and profiles, respectively) were determined by means of a confocal laser scanning microscope (CLSM) of the type LEXT OLS 4000 (Olympus, Hamburg, Germany) for every sample three times at random areas. A constant optical magnification (50×) was utilized leading to a scan area of 256 × 256 µm. The resulting scans have a resolution of 1024 by 1024 pixels. The software OLS4000 (Olympus, Hamburg, Germany) was used for data calculation and visualization. For detailed images, a scanning electron microscope (SEM) StereoScan360 (Cambridge Instruments, Cambridge, UK) was used.

### 2.4. Wetting Properties

To determine the resulting contact angle of the investigated surfaces after 1 and 7 days, a computer-controlled contact angle meter OCA 40 Micro (DataPhysics Instruments GmbH, Filderstadt, Germany) was used. The drop volume of distilled water was adjusted to 5 μL at a dosage rate of 1 μL/s. For each surface and time, the contact angle was measured three times.

### 2.5. Cell Culture

To study the cell adhesion, morphology, and growth on laser-structured surfaces compared to the reference, the human osteoblast cell line MG-63 (ATCC^®^ American Type Culture Collection CRL-1427™, Manassas, VA, USA) was used. In our previous work we could demonstrate that cell adhesion, spreading, and proliferation were stable over passages 5–30, and were similar to human primary osteoblasts [24]. The MG-63 osteoblasts were cultured in Dulbecco’s modified Eagle’s medium (DMEM) with 10% fetal calf serum (both Merck, Darmstadt, Germany) and 1% gentamicin (Ratiopharm GmbH, Ulm, Germany) at 37 °C and 5% CO_2_ atmosphere (incubator, SANYO CO2 INKUBATOR MCO-18AIC-UV, Panasonic Biomedical, Osaka, Japan). For the study, laser-structured and planar reference Ti6Al4V surfaces were exposed to cell culture immediately after wettability characterizations (1 and 7 days). The osteoblasts were seeded onto the samples (1 × 1 cm) at a density of 30,000 cells for 24 h.

### 2.6. Morphology and Spreading of MG-63 Cells

The morphology of MG-63 osteoblasts was analyzed by field emission scanning electron microscopy (FE-SEM, 5 kV; Merlin VP compact, Carl Zeiss, Oberkochen, Germany). The cells were fixed with 2.5% glutaraldehyde (Merck), dehydrated through a grade series of ethanol (30%, 50%, 75%, 90%, 100%), and dried in K850 critical point dryer (Emitech, Taunusstein, Germany). As the final step, cells were sputtered with a 20 nm gold-layer (SCD 004, BAL-TEC, Balzers, Liechtenstein, Liechtenstein). To image the cells, a high efficiency secondary electron detector (HE-SE) was used.

The cell spreading was analyzed by using ImageJ (Version 1.51f, Wayne Rasband, National Institutes of Health, Bethesda, MD, USA) of the FS-SEM images. For this, 40 cell areas per sample were calculated, and the statistical analyses were performed with GraphPad Prism (Version 7.02, GraphPad Software Inc., La Jolla, CA, USA) by non-parametric Kruskal–Wallis test post hoc uncorrected Dunn’s test (analysis of variance) (* *p* < 0.001). The data were presented as mean ± standard error of the mean (s.e.m.).

### 2.7. Actin Cytoskeleton Organization

The actin cytoskeleton organization of MG-63 cells was conducted by confocal laser scanning microscopy (CLSM) (LSM780, Carl Zeiss Microscopy GmbH, Jena, Germany) with helium-neon laser (excitation: 543 nm), and 40× water objective. Cells were fixed with 10% paraformaldehyde (PFA, Merck KGaA, Darmstadt, Germany), permeabilized with 0.1% Triton X-100 (4-(1,1,3,3-Tetramethylbutyl)phenyl-polyethylene glycol, Merck KGaA, Darmstadt, Germany) and incubated with phalloidin TRITC (phalloidin-tetramethyl-rhodamine, 1:7, Sigma-Aldrich, St. Louis, MO, USA). Next, the samples were embedded in Fluoroshield™ with DAPI (4′,6-Diamidin-2-phenylindol, DAPI, Sigma-Aldrich, St. Louis, MO, USA) on a cover slip and stored in the dark at 4 °C. For image acquisition, the ZEN 2011 software (ZEISS Efficient Navigation, ZEN 2011 SP4, black edition, Carl Zeiss, Carl Zeiss Microscopy GmbH, Jena, Germany) was used.

Furthermore, to obtain quantitative information of cytoskeletal organization, the actin filament number, length, and orientation were analyzed by using FilaQuant software (Institute of Mathematics, Mathematical Optimization, University of Rostock, Rostock, Germany) based on confocal images [25,26]. The mathematical image processing was completed for 10 cells per sample, and statistical evaluation was done using GraphPad Prism (Version 7.02, GraphPad Software Inc., La Jolla, CA, USA) by parametric ANOVA post hoc Bonferroni (analysis of variance) (* *p* > 0.05). The results were presented as mean ± s.e.m.

## 3. Results and Discussion

### 3.1. Surface Characterization

**Figure 1:**
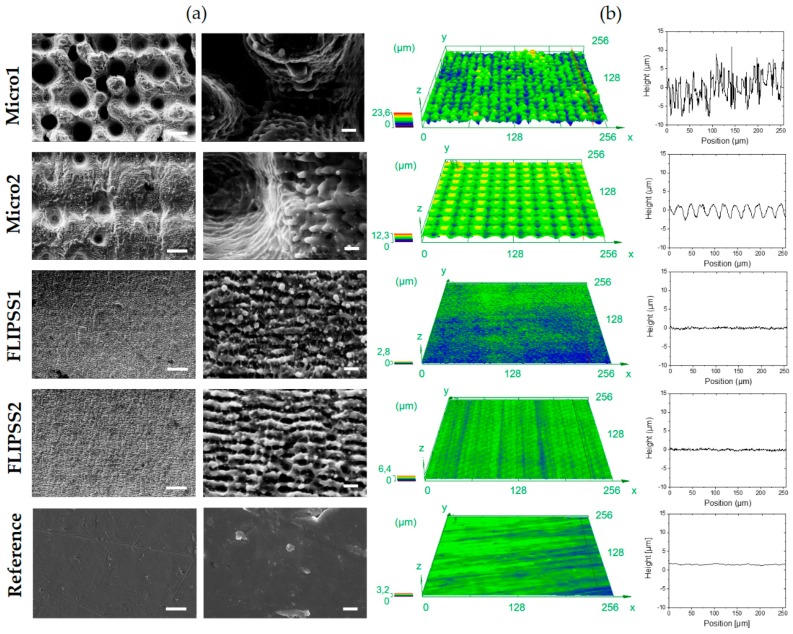
Surface characterization of laser-structured Ti6Al4V specimens. (**a**) Scanning electron microscope images (StereoScan360), scale bars left: 10 µm, right: 1 µm and (**b**) confocal laser scanning microscope images (LEXT OLS 4000), square side length: 256 µm, with corresponding elevation profile of laser irradiated structures and polished Ti6Al4V reference.

The SEM images with related CLSM images and elevation profile are shown in Figure 1. Generally, three types of structures can be observed: Stochastic pillared microstructures (Micro1), sinusoids periodic microstructures (Micro2) and nanostructures (FLIPSS1 and FLIPSS2). Under laser irradiation at 5 and 15 µJ, femtosecond laser-induced periodic structures (FLIPSS) are formed with a corresponding periodicity close to the laser wavelength (λ = 1030 nm). It is widely understood that the formation of these structures is caused by interferences between linearly polarized laser light and excited surface plasmon polaritons and orientated perpendicular to the polarization vector of the incident light [27]. Fluences are slightly higher than the ablation threshold resulting in a generation of laser-induced periodic structures (LIPSS). 

The resulting surface roughnesses (Sa) are shown in Table 2. It can be seen that the roughness varies in a range from Sa = 0.13 µm to a maximum roughness of 2.96 µm. The roughness Sa of the structures FLIPPS1 and FLIPPS2 are nearly the same or marginally higher than the roughness of the untreated reference. The small increase of Sa of the FLIPSS structures in relation to the reference is a result of the higher periodicity of the micro elevations, since the average height of the samples is almost similar. With increased pulse energy, microstructures with dual scale roughness are formed. Micro1 are stochastic structures due to high line overlap, Micro2 are sinusoids periodic structures caused by small line overlap from laser treatment. The formation of stochastic structures like Micro1 is discussed widely and attributed to different mechanisms. Thus, for example, hydrodynamic effects [28], varying ablation threshold through inhomogeneous element distribution or impurities in the surface of the material or inconsistent flow of the surface melt from previous irradiations appears to be responsible for the formation of these structures [29]. Figure 1 reveals that Micro2 structures are covered with more nanostructures than Micro1. FLIPPS are also created at higher pulse energies, since the fluence by a Gaussian laser beam decreases from the center to the edge of the irradiated area [30]. Due to the lower line overlap at similar number of overscans, FLIPPS are more extended on Micro2, because more area is primary irradiated with fluences slightly above the ablation threshold.

### 3.2. Wetting Properties of Ti6Al4V Surfaces

The resulting contact angles on the structured surfaces and the references are shown in Table 3. Directly after laser treatment, a strong hydrophilic behavior can be observed, especially on FLIPPS1, whereas the contact angles of FLIPPS2 samples are in the same order of the contact angles of the references. This could be caused by the different impact of the chemical composition of the surface due to laser irradiation with lower pulse energy and lower line overlap. It is supposed that laser treatment and subsequent exposition to air leads to a formation of TiO_2_/Al_2_O_3_ and unsaturated chemical compounds on the surface; this is associated with a hydrophilic behavior and leads to hydrophilic surfaces [31]. This mechanism appears to depend on the laser fluence, or rather the irradiated energy on a spot seems to determine the level of oxygen on the surface [11]. Otherwise, the organization of the laser-induced periodic structures could have an important impact on the wettability, since the roughness Sa of FLIPPS1 and FLIPPS2 are nearly the same but do obviously lead to very different contact angles. As shown in Figure 1, FLIPPS structures on the FLIPPS2 sample are stronger pronounced and distributed more homogeneous that could result in a stronger hydrophobic behavior. Further experiments and chemical analyses (for instance, X-ray photoelectron spectroscopy (XPS)) can help further our understanding. Generally, a clear increase of the contact angle over time (1 and 7 days) on all laser-structured surfaces can be observed. Explanatory approaches are given in different studies. Kietzig et. al. [11] attribute the phenomenon to the fact that the amount of carbon on the surface increases due to the composition of CO_2_ and the accumulation of nonpolar carbon on the surface. Long et. al. [20] reported an absorption of organic compounds from the ambient atmosphere onto the oxide surface, which leads to a more hydrophobic surface. A strong change in wetting behavior is also observed on the structures Micro2. The sample Micro2 is highly covered with periodic nanostructures, as mentioned before, that can be seen as a reason for the higher increase of the contact angles compared to Micro1 over time. It is known that (microscale-) roughness leads to an increased hydrophobic behavior on already hydrophobic surfaces [32].

### 3.3. Cellular Response

**Figure 2:**
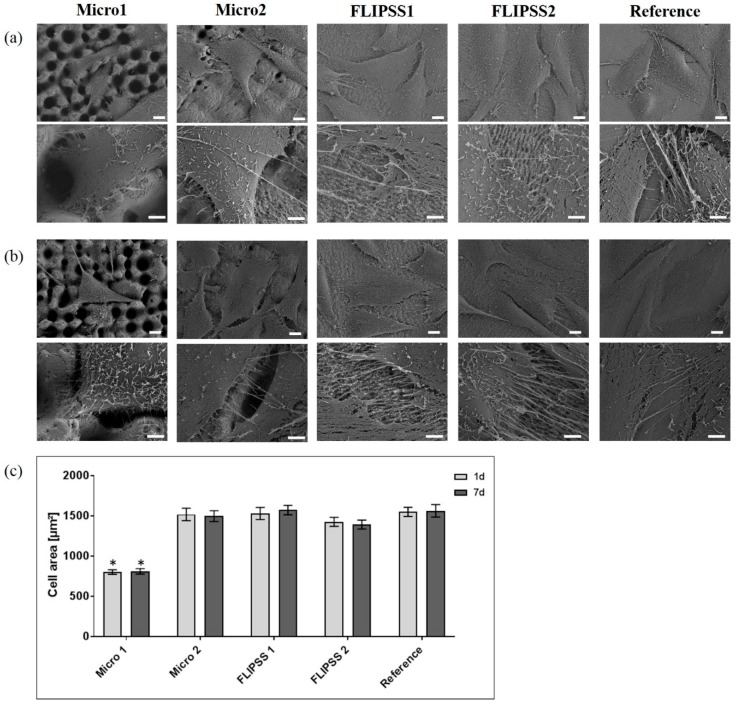
Cell morphology after 24 h on laser-structured samples (**a**) 1 day after laser process, and (**b**) 7 days after laser process (FE-SEM Merlin VP compact, scale bars above: 10 µm, below: 2 µm). (**c**) Cell area of osteoblasts on various nano/micro textured samples compared to planar reference (mean ± s.e.m., Kruskal–Wallis test post hoc uncorrected Dunn´s test, * *p* < 0.001, n = 40 cells). Note that the cell growth was impaired on Micro1, which is independent of the wettability (see Table 3).

The present in vitro study revealed no cytotoxic effect due to the femtosecond laser processes. The nano/micro textures produced by laser processes affected the adhesion, morphology, and growth of MG-63 cells. On Micro1 samples the cell morphology (Figure 2a,b) and the determined cell area (Figure 2c) were significantly different to the values on Micro2, FLIPPS1, FLIPPS2, and the reference. This cell behavior was independent of the surface wettability (after 1 and 7 days). In general, on the sample Micro1 fewer cells were detectable, embedded in deep cavities of the microstructure. It was also evident that the cells aligned with the structures due to the elevations and therefore could not spread out (Figure 2). Due to the laser micro texturing (Sa > 2.6 μm), some cells were in cavities or tried to spread along the structures. However, the cells could not completely align and spread well as they did on a smooth surface (FLIPSS1, FLIPPS2, and reference), but are limited and directed in their spreading and migration due to the size and shape of the microstructures. In these cases, the cells followed the structure of the surface and lined the elevations and craters. On all other surfaces more cells were observed. The osteoblasts on FLIPPS1 and FLIPPS2 as well as on the reference adhered tightly to the surfaces, and the spreading was superior. Cells on sample Micro2 were similar in morphology and spreading to reference and FLIPPS-structures, but with respect to their adhesion, it could be shown that they did not adhere tightly but rather spanned over sinusoids periodic microstructures.

The result with a reduced cell area on the stochastic pillar microstructure Micro1 is in agreement with another study which could show a significantly reduced cell spreading on regular pillar structures with sharp corners in the dimension of 5 µm [15]. It is known that the structuring of separate areas of the metallic surfaces can be used to cause a desired and particular cell behavior [33,34].

**Figure 3:**
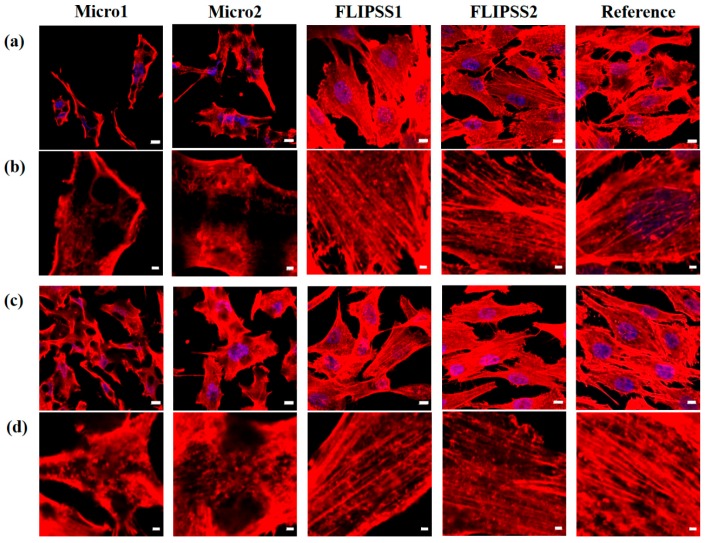
Organization of the actin cytoskeleton in MG-63 cells cultivated for 24 h on nano/micro textured samples. (**a**,**b**) 1 day after laser structuring, and (**c**,**d**) 7 days after laser structuring (LSM780, (**a**,**c**) zoom1, scale bars: 10 µm, (**b**,**d**) zoom4, scale bars: 2 µm; red: actin, blue: nucleus). Note that the actin cytoskeleton was only cortically arranged or in short filaments inside the cells on Micro1, in contrast to all other samples, where cells exhibit long filaments through the cells.

Another cell morphological aspect is the observation of the actin cytoskeleton. The CLSM images (Figure 3) confirmed the results of FE-SEM about the specific cell response on laser structures—the impaired cell growth on Micro1. The examination of actin cytoskeleton on Micro1 revealed only cortical actin fibers and short fragments within the cells. On the other laser-structured surfaces, cells formed a well-developed actin cytoskeleton with long filaments inside the cell.

On Micro2, due to the sinusoid periodic structures, actin was strongly cortically organized, and thin long filaments spanned the entire cell body. To supplement the qualitative confocal images (Figure 3), the actin filaments were quantified using the software FilaQuant [25,26]. The focus was on the total number of actin filaments, the length (total, max., mean), and the orientation of the cytoskeleton fibers. The quantification reveals a decrease of actin filament number of MG-63 cells on Micro1 compared to FLIPSS1 (7 days after laser processing), as well as FLIPSS2, and the reference (1 day after laser processing) due to the few cortical actin filaments (Table 4). Additionally, on micro textured samples (Micro1 & Micro2) a reduced total filament length could be quantified. The actin filament distribution indicated no preferred orientation in the MG-63 cells on the samples (0°: distinct one prefers direction; 28.65° homogenous distribution). Thus, a contact guidance could not be determined here. The cell spreading, morphology, and actin organization on FLIPSS1 and FLIPSS2 were comparable with the plane reference. The differences in cell behavior between the laser-structured samples correlate well with the size and shape of the nano- or microstructures, but not with the wettability. The differences in wettability after the sample’s storage time 1 and 7 days after laser treatment had no influence on the cell behavior as well.

The literature postulates that physico-chemical surface characteristics such as roughness and wettability influence cell adhesion, spreading, proliferation, and differentiation [2,3,9,35]. Raimbault et al. [21] pointed out that cell behavior is not necessarily positively modulated by hydrophilic surfaces, which is in accordance with our observations. Basically, we could not detect any cytotoxic effects on the femtosecond laser nano/micro textured samples. Kunzler et al. [34] could show that rat osteoblasts prefer rougher part on surface with gradually increasing surface roughness. The influence on osteoblast adhesion depended on the current size and depth dimensions as well as the roughness (Sa). Cells on FLIPSS nanostructures with low roughness (Sa < 0.15 µm) showed a phenotype similar to the reference structure. Due to increased pulse energy, the microstructures Micro1 and Micro2 influence the cell response. Due to the sinusoids periodic microstructure (Micro2) only slight impairments in cell adhesion and actin organization were detectable, but the morphology and spread were unaffected and comparable to the nanostructures FLIPSS and reference. On Micro1 with pronounced pillar profile dimension (depth: 9.57 ± 1.15 µm, width: 13.59 ± 1.23 µm), a clear impact on cell adhesion and growth was evident. In the literature, an increase in the roughness is related to an improved cell adhesion and growth behavior [2,13,36,37,38,39,40,41]. However, with increasing roughness in a microscale area, the contact between the cells and the surface is reduced, thus impairing the cell adhesion [2]. Belaud et al. [36] demonstrated in their work that the combination of nanoscale and curved structures stimulates the adhesion and migration of stem cells. The group of Boyan et al. [37,38,39,40] showed that osteoblasts are sensitive to micro/nano structures and the surface roughness has a clear impact on signal protein synthesis, local factor production, and finally on cell proliferation and differentiation. Dowling et al. [41] demonstrated a reduced cell area at a high surface roughness (Ra > 2365 nm) of plasma-treated polystyrene (PS) on MG-63 osteoblasts. In agreement with these findings, it was shown in the present study that a lower spreading potential of MG-63 cells occurs at higher surface roughness (Micro1, Sa > 2.6 µm) compared to the reference (Sa < 0.9 µm). However, there are inconsistencies between the present study and the findings of Dowling et al., especially concerning the effect of wetting on cell behavior. By comparing surface roughness/texture with the wettability, the present study showed that the surface wettability displayed no significant influence on cell morphology and spreading. Dowling et al. indicated the best cell adhesion at a contact angle value about 60° (best 64°). Also, other groups could show that wettability of material surfaces seems to be optimal for cell responses, with a moderate hydrophilic water contact angles (WCA) region between 45–68° [2,42,43].

Here, the osteoblast cell area was impaired on Micro1 due to the micro texture and not due to wettability. Another aspect of surface characterization needs to be explored in the next study, as it is known from the literature that surface energy affects the cellular response. Gentleman et al. [44] described the influence of surface free energy as an important part in osteoblast interaction at the interface to materials. Also, Hallab et al. [45] indicated that the surface energy may be more important for fibroblast adhesion than surface roughness. Moerke et al. [46] pointed out that a positive surface charge on microgrooves seemed to be an important parameter for the cellular outcome. 

In the end, the femtosecond laser nano/micro texture appears to be a good tool for producing topographically functionalized material surfaces to guide the adhesion and growth of bone cells [47]. Further studies could be able to show that the laser structuring of Ti6Al4V samples, which, apart from well suited to the mechanical, (electro) chemical, and biological properties of the material, can control the in vitro cellular behavior [13] and increase the osteointegration and consequently the durability of implants in vivo [48]. Furthermore, the first step in cell adhesion mechanism—protein adsorption—should be investigated. A possible reason for the equal cell adhesion on uncoated laser textures, after 1 and 7 days with different wettabilities, is the similar adsorption of protein layer on these surfaces [49]. Hasan et al. [50,51,52] showed that the protein absorption is a function of the hydrophobicity of surfaces that were chemically modified via salinization. It is assumed that the presence of FBS (fetal bovine serum) on a surface affects the cell adhesion. The effect of femtosecond laser structuring on protein adsorption and cell adhesion could be also be investigated in further studies.

## 4. Conclusions

Human MG-63 osteoblast growth is dependent on the nano/micro textures of femtosecond laser-structured titanium alloy. Microstructures with a pronounced pillar micro-profile (Micro1) showed a clear impairment on cell adhesion and spreading. The cellular response correlates well with the topology (size and shape) of the nano/micro texture, but not with the wettability. Laser processes are well suited for roughening titanium surfaces and thus modifying biomaterials in regenerative medicine. 

## Figures and Tables

**Table 1:** Laser parameter settings and procedure of laser scanning.

Structure	Laser Parameters			
	Pulse Energy (µJ)	Fluence in Focus (J/cm²)	Line Overlap (%)	Procedure of Laser Passages
Micro1	50	9.82	60	Grid
Micro2	50	9.82	30	Grid
FLIPSS1	15	2.95	90	Line
FLIPSS2	5	0.98	80	Grid

**Table 2:** Average area surface roughness Sa and surface micro-profile of laser-structured surfaces and reference. The values are listed separately for each specimen.

Sample	Roughness	Pillar/Profile Dimension	
	Sa (µm)	Depth (µm)	Width (µm)
Micro1	2.65 ± 0.19	9.57 ± 1.15	13.59 ± 1.23
	2.69 ± 0.10		
Micro2	1.09 ± 0.02	4.26 ± 0.32	25.13 ± 0.99
	1.13 ± 0.04		
FLIPSS1	0.14 ± 0.01	0.52 ± 0.05	–
	0.13 ± 0.004		
FLIPSS2	0.14 ± 0.01	0.50 ± 0.02	–
	0.15 ± 0.01		
Reference	0.08 ± 0.02	0.48 ± 0.02	–
	0.09 ± 0.01		

**Table 3:** Contact angle results after 1 and 7 days after laser treatment. The values are listed separately for each specimen.

Sample	Contact Angle (°)	
	Storage Time 1 Day	Storage Time 7 Days
Micro1	40.97 ± 1.01	73.53 ± 1.14
	39.93 ± 1.59	79.60 ± 2.5
Micro2	40.30 ± 3.16	98.60 ± 2.69
	43.73 ± 1.41	100.13 ± 4.42
FLIPSS1	18.80 ± 5.16	55.67 ± 4.27
	~ 0	59.70 ± 3.75
FLIPSS2	78.00 ± 1.94	92.50 ± 4.49
	75.17 ± 1.35	81.80 ± 5.06
Reference	88.80 ± 1.80	83.73 ± 2.09
	89.37 ± 1.91	89.20 ± 2.03

**Table 4:** Quantitative analysis of the actin cytoskeleton organization was performed using the FilaQuant software (confocal images in Figure 3); *(mean ± s.e.m.; ANOVA post hoc Bonferroni, n = 10 cells; n.s. between 1 day vs 7 days laser process).

Sample	Actin Filament Number	Total Filament Length (µm)	Mean Filament Length (µm)	Max. Filament Length (µm)	Orientation Dispersion (°)
**Storage time 1 day**					
Micro1	10.9 ± 2.9 ^a,b,c^	92.5 ± 38.5 ^a^	4.5 ± 0.7 ^a^	23.4 ± 9.4	17.5 ± 1.7
Micro2	20.2 ± 4.3	105.4 ± 21.3 ^a^	5.6 ± 1.1 ^a^	18.1 ± 6.6	22.8 ± 2.2
FLIPSS1	56.2 ± 14,6	387.5 ± 120.3	8.1 ± 1.1	33.5 ± 7.1	22.5 ± 2.0
FLIPSS2	49 ± 8.9	359.2 ± 43.9	7.8 ± 1.4	39.4 ± 8.3	21.8 ± 1.3
Reference	49.5 ± 8.6	552.1 ± 89.5	10.4 ± 0.2	52.5 ± 10.7	21.5 ± 1.7
**Storage time 7 days**					
Micro1	18.1 ± 3.2 ^b^	105.5 ±24.7 ^a,c^	3.7 ± 1.4	17.0 ± 5.6	24.5 ± 1.0
Micro2	21.7 ± 2.6	104.4 ±11.9 ^a,c^	3.6 ± 1.2	15.5 ± 4.0	22.0 ± 1.7
FLIPSS1	40.4 ± 4.1	213.7 ± 25.8	7.4 ± 4.3	40.3 ± 7.1	21.7 ± 2.1
FLIPSS2	35.8 ± 5.1	427.5 ± 43.9	6.4 ± 3.7	46.5 ± 6.1	20.4 ± 1.3
Reference	35.7 ± 7.4	552.1 ± 89.5	6.7 ± 3.9	47.4 ± 13.3	22.4 ± 2.2

^a^: * *p* < 0.05 vs. Reference; ^b^: * *p* < 0.05 vs. FLIPSS1; ^c^: * *p* < 0.05 vs. FLIPSS2.
